# A Method for Formulizing Disaster Evacuation Demand Curves Based on SI Model

**DOI:** 10.3390/ijerph13100986

**Published:** 2016-10-10

**Authors:** Yulei Song, Xuedong Yan

**Affiliations:** MOE Key Laboratory for Urban Transportation Complex Systems Theory and Technology, School of Traffic and Transportation, Beijing Jiaotong University, Beijing 100044, China; 14120879@bjtu.edu.cn

**Keywords:** evacuation demand curves, social influence, Susceptible-Infective model, sensitivity analyses, Tianjin Explosions

## Abstract

The prediction of evacuation demand curves is a crucial step in the disaster evacuation plan making, which directly affects the performance of the disaster evacuation. In this paper, we discuss the factors influencing individual evacuation decision making (whether and when to leave) and summarize them into four kinds: individual characteristics, social influence, geographic location, and warning degree. In the view of social contagion of decision making, a method based on Susceptible-Infective (SI) model is proposed to formulize the disaster evacuation demand curves to address both social influence and other factors’ effects. The disaster event of the “Tianjin Explosions” is used as a case study to illustrate the modeling results influenced by the four factors and perform the sensitivity analyses of the key parameters of the model. Some interesting phenomena are found and discussed, which is meaningful for authorities to make specific evacuation plans. For example, due to the lower social influence in isolated communities, extra actions might be taken to accelerate evacuation process in those communities.

## 1. Introduction

The past decades have witnessed an increasing number of disasters which have caused huge losses of life and property [1]. Hurricane Katrina in 2005 resulted in more than 10.5 billion dollars of damage. The WenChuan earthquake killed around 70 thousand people and caused direct economic losses of 8451 billion Yuan. To prevent or at least minimize the losses, an efficient evacuation plan is regarded to be crucial [2]. In the whole process of evacuation planning, the evacuation demands estimation is considered a key step. Lacking an accurate prediction of the evacuation demands, large traffic congestions might happen in disaster evacuations [3,4]. In general, the times at which people begin to evacuate can be expressed as an evacuation response curve or an evacuation demand curve [5,6,7]. However, it is hard to predict evacuation demand curves since the individual evacuation-decision making (whether and when to evacuate) is influenced by many factors (i.e., individual characteristics, location, and social influence). In fact, it is regarded as a socio-psychological process and may be easily influenced by other people, which is called a “herd behavior” [8]. In spite of the qualitative insights from many social science studies about the impact of social influence on socio-psychological process, such as epidemics spreading, rumors spreading, and diffusion of innovation, models formulizing evacuation demand curves with the perspective of social contagion in decision making are lacking [9]. This study aims to develop a model taking into account both social influence and other important factors like individual characteristics, location, and warning degree to formulize the disaster evacuation demand curves.

### 1.1. About the Disaster Evacuation Demand Curve

The disaster evacuation demand curve is used to describe the time distribution of evacuation demand. The point of the curve represents the cumulative evacuation demand as a function of time. Predicting this curve is regarded as an important step for the disaster management authorities to make an evacuation plan. However, it is not an easy job to predict this curve, as the individual evacuation decision making is a complex process which is influenced by many aspects [10]. Fortunately, previous studies have focused on the relationship between the individual decision making and influencing factors, which can be roughly summed up in four types: individual characteristics, social influence, geographical location, and warning degree. It was found that the evacuation decision making was related with individual characteristics like gender, race, age, or children in the household [11,12,13]. Specifically, the families with a child or elder were considered to be more likely to leave their home when they are exposed in risk [14,15]. Similarly, home ownership, the number of individuals in the household, and income levels were found to be significantly related with mobilization times, which were regarded as an important component of the time of departure for the evacuees [16]. Moreover, the individual risk perception was argued as an important factor affecting evacuation decision making and people who have perceived a high risk prefer to leave [15,17]. Besides individual characteristics, the geographical location was reported to affect the evacuation decision [18]. It was found that people who live close to threats or have access to evacuation resources were more likely to evacuate [5,19,20]. Additionally, the evacuation warning was found to be an important variable affecting public response and those who receive the warning information in the risk areas are more than twice as likely to evacuate [15]. The warning information (like wording and content of evacuation orders) can influence not only the evacuation demand, but also the urgency at which people leave [21,22].

### 1.2. Social Influence on Individual Evacuation Decision

In recent studies, researchers began to emphasize the role social influences played in individual evacuation decision making [23,24,25]. In social psychology, people′s emotions, opinions, or behaviors are easily influenced by others, which can be expressed as herd behavior. Herd behaviors were found to occur frequently in everyday decisions based on learning from the information of others [8,26,27]. There is no exception in the individual evacuation decision making. Evidence was found in previous studies demonstrating that social influence plays a key role on the decision to evacuate or not [24,28,29,30,31]. This social influence is always from people you contact like friends or relatives. Without enough information about the disaster, people’s risk perceptions are always based on the evacuation decisions of others that they contact [21]. Moreover, people are more likely to evacuate when they find most of friends or relatives contacted have decided to leave even if their initial response were to remain [14]. With social influence, in many actual evacuations, it has been observed that a large number of people in low risk areas evacuated along with evacuees from high risk areas, which is often referred as shadow evacuation [32,33].

To describe the role of social influence in socio-psychological processes, Susceptible-Infective (SI), Susceptible-Infective-Recovered (SIR), and Susceptible-Infective-Susceptible (SIS) models used several differential equations to depict the social influence in disease spread process [34,35]. Daley and Kendall (DK) model and Maki and Thompson (MT) model used several simple multiplication formulas to formulize the rumor spreading process with social influence [36,37]. Threshold, cascade, and voters models were used to describe the diffusion of innovation. However, studies developing models with social influences to explicitly depict evacuation decision making process are lacking [2,9,25].

### 1.3. Disaster Evacuation Demand Curve Modeling

Numerous studies have been focused on the topic of evacuation-plans modelling and models’ validation issues [17], models for evacuation demand curves are relatively few. Early approximations of demand curves were based on empirical evidence, stated intention surveys, planner judgment, and simulation of the warning information diffusion [5,38]. In the 1980s, S-curves (named for their sigmoid shape) were used to predict evacuation demand curves [39,40]. In the S-curves, the evacuation rate (the percentage of people who choose to evacuate) starts slowly, then increase rapidly, and finally slow and gradually close to zero. Based on behavioral analyses of past disasters, the S-curves can be classified as rapid, medium, and slow types [41]. With the advantages of mathematically simple expression and roughly matching the data from a typical evacuation, the S-curves have been widely used by researchers [41,42]. Nevertheless, some disadvantages also exist in S-curves: it is too simplistic to formulize a mechanism of people’s evacuation decision making toward disaster and the selection of demand curve (rapid, medium, and slow types) is subjective [43].

To remedy the drawbacks in S-curves, a sequential logit model based on empirical data was proposed in 2004 [43,44]. In 2007, Fu et al. [45] selected several predictor variables for logistic regression and calculated corresponding coefficients using data from Hurricane Floyd. Although the sequential logit model considers characteristics of disasters and geographic locations, it is hard to effectively explain the significant power of social influence on individual behaviors toward the evacuation decision.

Recently, with social science studies increasing, some researchers began to describe the evacuation demand curves in the view of social science. A threshold model was used to describe the evacuation decision making process [25]. Moreover, a probabilistic model incorporating both individual characteristics and social influence were also proposed based on a social network [2]. These studies focused on people’s evacuation decision making at individual levels through an underlying social network and how solutions would be more difficult to get when a large population was considered as the subject in models.

Overall, models are lacking that can fundamentally formulize an evacuation decision making process influenced by many factors, especially by social influence.

### 1.4. Motivation and Objective of This Study

In this paper, we aim to develop a model to mathematically depict the disaster evacuation demand curves with the perspective of social contagion of decision making and discuss how the potential factors influence the curves. Inspired by social influence mechanisms of SI model in the epidemic-spreading process, an improved model with an emphasis of social influence is developed, which can also effectively capture other factors for deducing the evacuation decision making process. The model is developed not at an individual level, but from an aspect of evacuation group and the group can be selected as a community or a set of people with homogeneous individual characteristics in a risk area. The evacuation demand curves in different groups are discussed in this paper. The rest of the paper is organized as follows. In Section 2, we develop the susceptible-infective model framework for disaster evacuation demand curves and formulize the effect of all factors on the curves. In Section 3, we propose a method for parameter sensitivity analyses. In Section 4, the Tianjin Explosion is used as a studying case for the model application. Section 5 and Section 6 are the conclusion and discussion, respectively.

## 2. Model Development for Evacuation Demand Curves Estimation Based on SI Model

### 2.1. Model Framework

**Figure 1:**
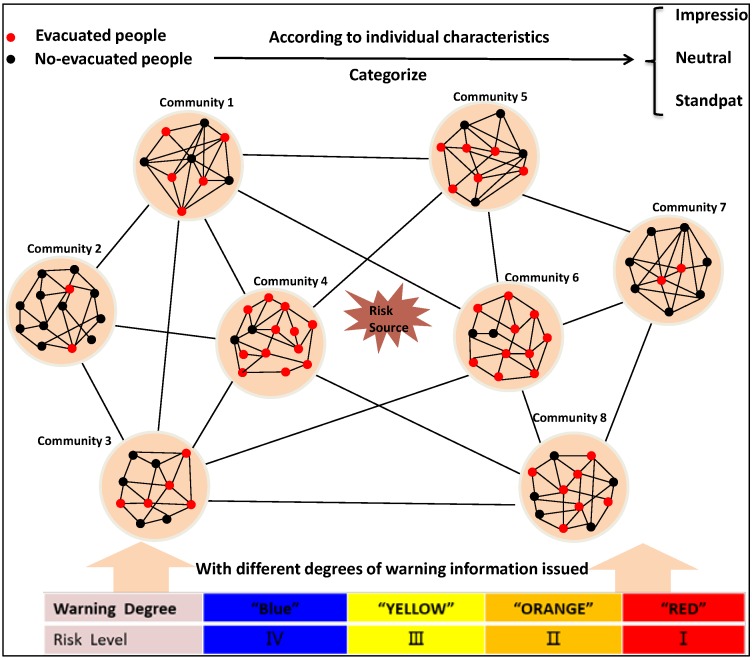
Four factors influencing individual evacuation decision making.

In this paper, the evacuation is supposed to be an irreversible monotone process and once an individual decides to evacuate he/she would remain evacuating. Based on whether to decide to evacuate or not, people can be in a corresponding state of either evacuated (have decided to evacuate) or non-evacuated (have not decided to evacuate). The evacuation decision making is supposed to be influenced by four factors: individual characteristics, social influence, geographical location, and warning degree as is shown in the Figure 1.

The SI model, a simple epidemic model, was initially created to explain the epidemic spreading process by Kermack, W.O. and McKendrick, A.G.A in 1927 [34]. In this model, a fixed population (*Q*) is taken into consideration with only two compartments: Susceptible and Infective. *S*(*t*) and *I*(*t*) represent respectively the percentage of “Susceptible” and “Infective” people at time *t*. It is assumed that every person contacts λ people every day and the susceptible people will be infected once contacted by infected people. The spreading process of epidemic diseases can be easily expressed:

(1) $$Q\frac{dI}{dt}=\lambda QSI\;\;\;\;\;\;S(t)+I(t)=1\;\;\;I(0)=I_{0}$$

It can be shown that Equation (1) is a logistic model and the solution is:

(2) $$I(t)=\frac{1}{1+(\frac{1}{I_{0}}-1)e^{-\lambda t}}$$

Inspired by social influence mechanisms of SI model in the epidemic spreading process, the herd behavior in the disaster evacuation decision making process can be described well by the SI model framework or a differential equation as there are many similarities between them. On the one hand, the contacts in epidemic spreading also happened in the evacuation decision making by an underlying social network. On the other hand, there are also only two states (evacuated and unevacuated) in the evacuation decision making process. With the four factors into consideration, the disaster evacuation decision making process can be described by a modified SI model that has changed or added additional variables as shown in Equation (3).

(3) $$\frac{dI}{dt}=\gamma \left[g_{1}+g_{2}+f_{1}+f_{2}\right]$$

where *I(t)* represents the total number of evacuated people at time *t*. The population is assumed to be fixed as *Q*. γ is the effective transformation rate which is assumed to be related only with individual characteristics. $g_{1}$, $g_{2}$, $f_{1}$, and $f_{2}$ respectively represent the social influence inside and outside of the community, geographical location, and warning degree. Similarly, γ $g_{1}$, γ $g_{2}$, γ $f_{1}$, and γ $f_{2}$ means increasing the number of evacuated people in unit time respectively by the four aspects above.

### 2.2. Formulization for Effect of Factors on Disaster Evacuation Demand Curves

#### 2.2.1. Individual Characteristic

The individual characteristics are one kind of important factor affecting the evacuation decision making. The individual characteristics have many aspects that can influence the evacuation decision like gender, age, individual risk belief, family member, income, having a car, knowledge about threat, evacuation experience, individual physical condition (like injuries present), and so on. To reflect the influence of individual characteristics, people are divided into three types (“Impressionable”, “Neutral”, and “Standpat”) in this paper. As the name implies, “Impressionable” means the people who are most easily influenced by social influence, geographical location, and warning degree during the process of evacuation decision making while “Standpat” is on the contrary. It should be pointed that there are many ways to categorize people in statistics or fuzzy mathematics. In this paper, a method of multiple discriminate analysis is applied in people categorization. Specifically, six variables (age, risk belief, family member structure, evacuation experience, income, and gender) were chosen as explanatory variables and “Impressionable”, “Neutral”, and “Standpat” were chosen as explained variables based on degrees of people’s evacuation willingness.

With the effect of individual characteristics, Equation (3) can be modified as Equation (4):

(4) $$\frac{dI}{dt}=\gamma^{k}\left[g_{1}+g_{2}+f_{1}+f_{2}\right]\;\;\;\;k=1,2,3$$

where $\gamma^{1}$, $\gamma^{2}$ and $\gamma^{3}$ respectively represent effective transformation rates for the “Impressionable”, “Neutral”, and “Standpat” types. Obviously, $\gamma^{1}>\gamma^{2}>\gamma^{3}$. It should be pointed that all parameters other than gamma in Equation (4) are assumed to be type-independent in this paper.

In this paper, it is assumed that there are *N* communities in risk areas. So the total people are divided into 3 × *N* groups. With the two evacuation states, the total people in the risk area can be expressed:

(5) $$Q=\sum_{j=1}^{N}Q_{j}=\sum_{k=1}^{3}Q^{k}=\sum_{j=1}^{N}\sum_{k=1}^{3}Q_{j}^{k}=\sum_{j=1}^{N}\sum_{k=1}^{3}\left(S_{j}^{k}+I_{j}^{k}\right)\;\;\;\;$$

(6) $$I=\sum_{k}I^{k}=\sum_{k}\sum_{j}I_{j}^{k}=\sum_{j}I_{j}$$

(7) $$S=\sum_{k}S^{k}=\sum_{k}\sum_{j}S_{j}^{k}\;=\sum_{j}S_{j}\;$$

where $Q_{j}^{k}$, $I_{j}^{k}$, and $S_{j}^{k}$ respectively represent the numbers of total, evacuated, and unevacuated people in type *k* in community *j*. $Q^{k}$, $I^{k}$, and $S^{k}$ respectively represent the number of total, evacuated, and unevacuated people in type *k*. $Q_{j}$, $I_{j}$ and $S_{j}$ respectively are the number of total, evacuated, and unevacuated people in community *j*. Function $I_{j}^{k}$,$I_{j}$, and I are the evacuation demand curves in respective groups.

#### 2.2.2. Social Influence

Given the social influence, it is obvious that an evacuee is more likely to evacuate when most of his neighbors or people he contacts decide to evacuate. In other words, the individual evacuation decision can be influenced by the present evacuation state of society. However, among all of the people who the evacuee may contact in any communities, the people who live in the same community may have a greater influence than those from other communities [46]. Based on this point, the evacuation state inside and outside community are both taken into consideration in this paper.

##### Evacuation State Inside the Community

As is shown in Figure 1, every community can be seen as a small network. Every person is like a vertex and the contact with other people inside community is like a side of the network. So the contact frequency of an individual per unit time in the community is like the degree of a node in the network. In a random graph, the node degrees have been traditional assumed to follow a Poisson distribution [47], which indicates that most nodes have approximately the same number of links (close to the average degreeλ ) as Equation (8).

(8) $$P(k)=\frac{\lambda }{k!}e^{-\lambda },k=0,1...$$

In a community, the contact frequency of an individual per time can also be regarded to follow a Poisson distribution. For simplicity, it is assumed to equal to $\lambda_{0}$ (the expectation of the Poisson distribution) in this paper.

With the effect of evacuation state inside the community, Equation (4) can be further modified as Equation (9):

(9) $$\frac{dI_{j}^{k}}{dt}=\gamma^{k}\left[\lambda_{0}\frac{S_{j}^{k}}{Q_{j}}I_{j}+g_{2}+f_{1}+f_{2}\right]$$

where $\lambda_{0}$ is the contact frequency of an individual per time and $\gamma^{k}\lambda_{0}\frac{S_{j}^{k}}{Q_{j}}$ means that if every evacuated people of Community *j* contacts $\lambda_{0}$ people of Community *j* over unit time, there are an average $\gamma^{k}\lambda_{0}\frac{S_{j}^{k}}{Q_{j}}$ unevacuated people (in Community *j* and type *k*) who will change their mind to evacuate.

##### Evacuation State Outside the Community

Obviously, contacts also exist between communities. In this paper, the contact frequency per time is assumed to be only related with the geographic distance between two communities. In general, there is a negative correlation between the contact frequency and the geographic distance, which can be described with a normal distribution probability density function in Equation (10).

(10) $$\;\lambda (d)=\lambda_{0}\frac{\alpha }{\sqrt{2\pi \sigma }}e^{-\frac{d^{2}}{2\sigma^{2}}}\propto \frac{1}{d}\;\lambda_{0},\alpha ,\sigma ,d\geq 0$$

where ∝ represents a correlation. For example, A ∝ B means A is in positive correlation with B and $A\propto \frac{1}{B}$ means A is negative correlation with B. So, $\lambda (d)\propto \frac{1}{d}$ means that λ(d) is negative correlation with *d.*

In addition to the contact frequency, the effect of other communities on an evacuation decision is also related with the risk level of those communities. In a simple example, an unevacuated resident may be more likely to evacuate when he receives a leaving message from an evacuated resident in a low-risk community than one in a high-risk community. Thus, a negative correlation is existed between the influence (*l*) of other communities and the risk level (*r*). Usually, the risk level (*r*) is negatively correlated with the distance (*d*) from the community location to the risk source. Therefore, the influence (*l*) of other communities should be positively related with the geographic distance (*d*), which can be substituted by a function h(d) in Equation (11).

(11) $$\begin{array}{l} l(r)\propto \frac{1}{r}\\ r(d)\propto \frac{1}{d} \end{array}\}\Rightarrow h(d)=l(r(d))\propto d$$

where l(r) is the influence function about risk level *r* and r(d) is the risk level function about the distance to the risk resource *d*, which derives the final influence functionh(d).

So the influence of the evacuation state outside the community on an evacuation decision can be described as Equation (12):

(12) $$\frac{dI_{j}^{k}}{dt}=\gamma^{k}\left[\lambda_{0}\frac{S_{j}^{k}}{Q_{j}}I_{j}+\frac{S_{j}^{k}}{Q_{j}}\sum_{k}\sum_{i\neq j}I_{i}^{k}\lambda (d_{i,j})h(d_{i,0})+f_{1}+f_{2}\right]\;=\gamma^{k}\left[\lambda_{0}\frac{S_{j}^{k}}{Q_{j}}I_{j}+\frac{S_{j}^{k}}{Q_{j}}\sum_{k}\sum_{i\neq j}I_{i}^{k}\lambda_{0}\frac{\alpha }{\sqrt{2\pi \sigma }}e^{-\frac{d_{i,j}{}^{2}}{2\sigma^{2}}}h(d_{i,0})+f_{1}+f_{2}\right]$$

where $d_{i,0}$ is the distance from community *i* to the risk source.

#### 2.2.3. Geographical Location

In addition to the contact frequency in Section 2.2.2, the geographical location also influences the risk level. In most disasters, the risk level is negatively related with the distance to the risk source. In other words, the further away from the risk source where a resident lives, the lower risk that he/she will receive. In this paper, without consideration of disasters changing over time, the effect of geographical location on the evacuation decision is assumed to be weakened. Based on assumptions above, the influence function of geographical location should decrease monotonically with time and the distance to the risk source as follows:

(13) $$\;f(d_{j,0},t)\propto \frac{1}{d_{j,0}},\frac{1}{t}\;\;\;\;j=1,2,...,N;\;\forall t$$

With the effect of geographical location, Equation (12) can be extended as below:

(14) $$\frac{dI_{j}^{k}}{dt}=\gamma^{k}\left[\lambda_{0}\frac{S_{j}^{k}}{Q_{j}}I_{j}+\frac{S_{j}^{k}}{Q_{j}}\sum_{k}\sum_{i\neq j}I_{i}^{k}\lambda (d_{i,j})h(d_{i,0})+S_{j}^{k}f(d_{j,0},t)+f_{2}\right]\;\;\;$$

the expression shows that $\gamma^{k}S_{j}^{k}f\;(d_{j,0},t)$ number of unevacuated people (in type *k* in community *j*) will change minds to evacuate per time influenced by the location at time *t*.

#### 2.2.4. Warning Degree

With the great power of warning information on evacuation demands, almost all of cities in the world have developed warning system to guide people evacuation under emergency [10]. The issue of evacuation warnings (like “blue”, “yellow”, “orange”, and “red”) can directly influence evacuation decision making. In most cases, people’s perceived risk levels are mainly from the warning degree. For example, a “red” warning degree means that people are more likely to be heavily threated by the disaster and their perceived risk level will be very high. In fact, there are many warning types and, in general, the types are not same in different countries. For an example, there are four warning levels (“blue”, “yellow”, “orange”, and “red”) for hurricane warnings in China; in America, there are five types including a “white” warning level. Those warning types are considered to be able to directly reflect the danger level of disasters and there are also other forms of warning types like various evacuation orders which can also reflect the danger level of disasters. An evacuation order that people within 5 km to the risk source are needed to evacuate can be seen as a higher degree of warning than one that people within only 1 km are need to evacuate. No matter what the warning types are, the role warning degree plays in individual evacuation decision making is the same. In general, like the injection effect in patients or the weakening social effect of many policies over time, we assume that the effect of evacuation warning is also weakened with time before the next degree of warning information coming. To describe the effect, we build an influence function of warning information, which is monotonically decreasing with time in every warning degree. When several different degrees of warning information were issued respectively in a disaster evacuation process, the influence function of warning degrees in this evacuation can be a piecewise function consisting of several monotonically decreasing influence functions of warning information.

**Figure 2:**
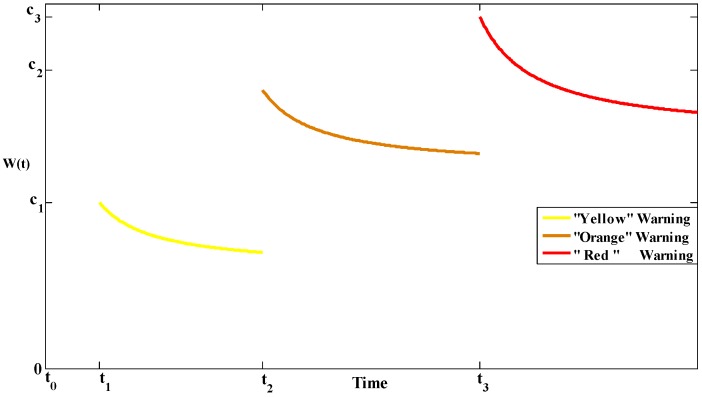
The influence function of the three warning degrees in this case.

For a case that three kinds of warning degrees are published at different instants of time (the “yellow”, “orange”, and “red” warning degrees are issued at $t_{1}$, $t_{2}$, and $t_{3}$ in sequence) in the process of an evacuation, the influence function of warning degree can be easily depicted in Equation (15). Equation (16) is used to reflect the different influences caused by the three degrees of warning information and Figure 2 shows an example for the degradation patterns in the effects of the three warning degrees over time.

(15) $$w(t)=\left\{\begin{array}{ll} b_{0} & 0\leq t<t_{1}\\ \frac{a_{1}}{t}+b_{1} & t_{1}\leq t<t_{2}\\ \frac{a_{2}}{t}+b_{2} & t_{2}\leq t<t_{3}\\ \frac{a_{3}}{t}+b_{3} & t_{3}\leq t \end{array}\right\}$$

(16) $$a_{1}<a_{2}<a_{3};\;b_{0}<\frac{a_{1}}{t_{1}}+b_{1}<\frac{a_{2}}{t_{2}}+b_{2}<\frac{a_{3}}{t_{3}}+b_{3}$$

In fact, the effect of warning degree on individual evacuation decision is always discussed with warning message dissemination technique. Warning dissemination is regarded to be achieved through both “formal systems” (from official warning through TV, radio, telephone, sirens, and door-to-door knocking) and “informal systems” (personal notification from neighbors, friends, and relatives) [48,49]. In this paper, we do not add a function to reflect the effect of warning message dissemination technique because this effect can be expressed in the function w(t) by changing the shape of the function.

### 2.3. The Complete Model for Formulizing a Disaster Evacuation Demand Curve

Taking into consideration of the four factors above, the complete model for formulizing disaster evacuation demand curves based on the SI model is shown as follows:

(17) $$\frac{dI_{j}^{k}}{dt}=\gamma^{k}\left[\lambda_{0}\frac{S_{j}^{k}}{Q_{j}}I_{j}+\frac{S_{j}^{k}}{Q_{j}}\sum_{k}\sum_{i\neq j}I_{i}^{k}\lambda (d_{i,j})h(d_{i,0})+S_{j}^{k}f(d_{j,0},t)+S_{j}^{k}w(t)\right]$$

k=1,2,3;j=1,2,...,N;∀t

(18) $$h(d)=l(r(d))\propto d\;\;\;f(d,t)\propto \frac{1}{d},\frac{1}{t}\;\;\;\lambda (d)=\lambda_{0}\frac{\alpha }{\sqrt{2\pi \sigma }}e^{-\frac{d^{2}}{2\sigma^{2}}}\;\;\gamma^{1}>\gamma^{2}>\gamma^{3}>0$$

(19) $$Q^{k}=\sum_{j}Q_{j}^{k}\;\;\;Q_{j}=\sum_{k}Q_{j}^{k}\;\;\;Q_{j}^{k}=S_{j}^{k}+I_{j}^{k}\;\;\;k=1,2,3;j=1,2,...,N;\forall t$$

(20) $$Q_{j}^{k},S_{j}^{k},I_{j}^{k},d_{i,j}\geq 0\;\;\;\;\;\;\;k=1,2,3;i,j=1,2,...,N;\forall t\;$$

(21) $$S_{j}^{k}(0)=S_{j,0}^{k}\;\;\;I_{j}^{k}(0)=I_{j,0}^{k}\;\;\;\;\;\;\;k=1,2,3;\;\;j=1,2,...,N$$

In fact, Equation (17) contains 3*N differential equations, which reveals the mechanism of the model. Constraints (18) define some necessary properties of functions like the warning influence function.

Unfortunately, it is hard to get the analytic solutions of Equation (17), though Function h(d) and f(d,t) are both easily depicted as a linear function. However, it is easy to get the numerical solution with an iterative algorithm.

## 3. Method for Parameter Sensitivity Analyses

Generally, a sensitivity analysis is a crucial step to study how the variation in the output of a model can be apportioned to different sources of variations. In this paper, the sensitivity analysis aims to examine the variation of total evacuation demand curve caused by the changing parameter values within a certain range. To describe the variation, the values of the curve in five moments ($t_{1}$, $t_{2}$, $t_{3}$, $t_{4}$, $t_{5}$) are used as output variables in this sensitivity analysis. So, the output values should be a five-dimensional vector. Equations (22) and (23) are the output with a set of parameters $\beta =(\beta_{1},\beta_{2},...,\beta_{m})$ and a set of basic reference parameters $\beta^{*}=(\beta_{1}{}^{*},\beta_{2}{}^{*},...,\beta_{m}{}^{*})$.

(22) $$\;O=P(\beta_{1},\beta_{2},...,\beta_{m})=(I_{\beta }(t_{1}),I_{\beta }(t_{2}),I_{\beta }(t_{3}),I_{\beta }(t_{4}),I_{\beta }(t_{5}))\;\;$$

(23) $$O_{*}=P(\beta_{1}{}^{*},\beta_{2}{}^{*},...,\beta_{m}{}^{*})=(I_{*}(t_{1}),I_{*}(t_{2}),I_{*}(t_{3}),I_{*}(t_{4}),I_{*}(t_{5}))$$

In this paper, the single factor sensitivity analysis is used and the output is shown in the Equation (24) with only one parameter changed:

(24) $$O_{\beta_{k}}(\beta_{k})=P(\beta_{1}^{*},...,\beta_{k-1}^{*},\beta_{k},\beta_{k+1}^{*},...,\beta_{13}^{*})=(I_{\beta_{k}}(t_{1}),I_{\beta_{k}}(t_{2}),I_{\beta_{k}}(t_{3}),I_{\beta_{k}}(t_{4}),I_{\beta_{k}}(t_{5}))$$

So, we define the sensitivity function (SF) using a dimensionless method:

(25) $$S(\beta_{k})\;≜\underset{\Delta \beta_{k}\to 0}{\lim}\left\|\frac{\Delta O_{\beta_{k}}}{O_{\beta_{k}}}\right\|\;/\;\left\|\frac{\Delta \beta_{k}}{\beta_{k}}\right\|=\underset{\Delta \beta_{k}\to 0}{\lim}\left\|\frac{O_{\beta_{k}}(\beta_{k}+\Delta \beta_{k})-O_{\beta_{k}}(\beta_{k})}{O_{\beta_{k}}}\right\|\;/\;\left\|\frac{\Delta \beta_{k}}{\beta_{k}}\right\|$$

where $S(\beta_{k})\;$ is the sensitivity value about parameter $\beta_{k}$ and a higher value indicates a higher sensitivity of model about the parameter. ‖.‖ is a norm in mathematics, which represents the size or length of the vector. In this paper, ‖.‖ is defined below, which is like the “Euclidean norm”.

(26) $$\left\|\frac{\Delta O_{\beta_{k}}}{O_{\beta_{k}}}\right\|=\left\|\frac{O_{\beta_{k}}(\beta_{k}+\Delta \beta_{k})-O_{\beta_{k}}(\beta_{k})}{O_{\beta_{k}}}\right\|≜({\left|\frac{I_{\beta_{k}+\Delta \beta_{k}}(t_{1})-I_{\beta_{k}}(t_{1})}{I_{\beta_{k}}(t_{1})}\right|}^{2}+{\left|\frac{I_{\beta_{k}+\Delta \beta_{k}}(t_{2})-I_{\beta_{k}}(t_{2})}{I_{\beta_{k}}(t_{2})}\right|}^{2}+{\left|\frac{I_{\beta_{k}+\Delta \beta_{k}}(t_{3})-I_{\beta_{k}}(t_{3})}{I_{\beta_{k}}(t_{3})}\right|}^{2}\;{+{\left|\frac{I_{\beta_{k}+\Delta \beta_{k}}(t_{4})-I_{\beta_{k}}(t_{4})}{I_{\beta_{k}}(t_{4})}\right|}^{2}+{\left|\frac{I_{\beta_{k}+\Delta \beta_{k}}(t_{5})-I_{\beta_{k}}(t_{5})}{I_{\beta_{k}}(t_{5})}\right|}^{2})}^{\frac{1}{2}}$$

Consequently, if we have a set of basic reference values of parameters and respective value ranges of parameters, we will get all of the parameter sensitivities of this model.

## 4. Case Study-Tianjin Explosions

In this section, the disaster event of Tianjin Explosions is used as a studying case. Without access to partial data, this case is mainly used to (a) illustrate numerical solution of this model; (b) analyze the effects of the four factors on the evacuation demand curves; and (c) perform the sensitivity analysis of parameters in the model.

### 4.1. Tianjin Explosions Description

The explosions occurred in a dangerous materials storage at about 11:30 pm (Beijing time) at the Port of Tianjin on 12 August 2015, which resulted in 173 deaths, 8 missing, and 798 non-fatal injuries. The first two explosions were occurred within 30 s, which were equal to about 24 tons of TNT exploding and fire—caused by the initial explosions—repeatedly caused secondary explosions. In the blasts, many chemicals leaked, like sodium cyanide, which can react with water to form highly toxic and flammable hydrogen cyanide gas. Out of consideration for risk of secondary explosions and toxic substances spreading, the local authority issued two evacuation orders. The first one, issued half an hour after the first two explosions happened, ordered people within one kilometer to evacuate. Sixty hours later, the second one was issued to order people within three kilometers to move out. Like a general evacuation, the shadow evacuation behaviors were also found in this evacuation namely that people, especially those living three to six kilometers from explosion source, also joined the evacuation.

### 4.2. Preliminary

**Figure 3:**
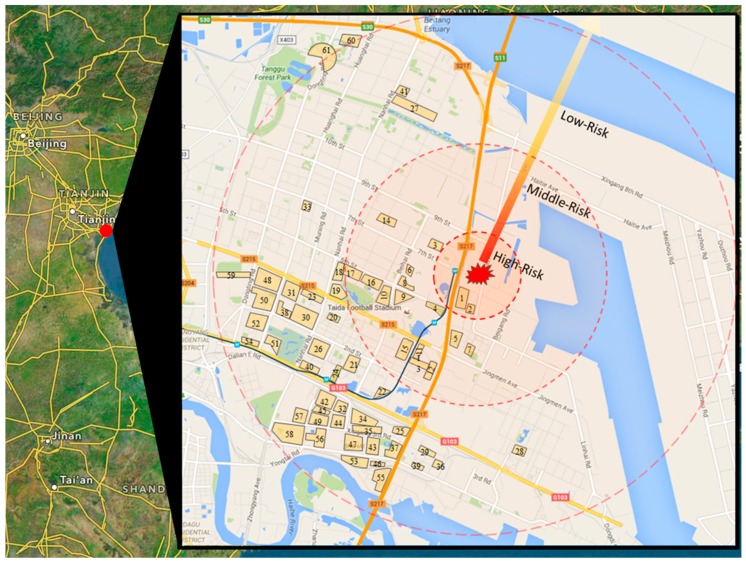
Maps of Tianjin explosions.

As is shown in Figure 3, there are 61 communities labeled as 1–61 according to the distance to the explosion source and the distance between every two communities can be calculated easily. There are totally around 115,350 residents living within six kilometers to the explosion source. It is assumed that people in every community are divided equally into three types according the individual characteristics. The two evacuation orders can be seen as two different degrees of warning information just like “orange” and “red” warnings, which issued at *t* = 0 and *t* = 60. Note that *t* = 0 represents the moment when the first evacuation order was issued rather than the moment of the first two explosions. In fact, *t* = 0 started at half an hour later after the explosions happened. In this example, Function h(d) is a simple linear function of distance *d*. Functions f(d,t) and w(t) are also expressed with simple elementary functions and the main model expression is described as follows:

(27) $$\frac{dI_{j}^{k}}{dt}=\gamma^{k}[\lambda_{0}\frac{S_{j}^{k}}{Q_{j}}I_{j}\frac{S_{j}^{k}}{Q_{j}}\sum_{k}\sum_{i\neq j}I_{i}^{k}\lambda_{0}\frac{\alpha }{\sqrt{2\pi \sigma }}e^{-\frac{d_{i,j}{}^{2}}{2\sigma^{2}}}(a_{1}d_{i,0}+b_{1})+S_{j}^{k}(\frac{a_{2}}{d_{j,0}{}^{2}}+\frac{b_{2}}{t+10})+S_{j}^{k}w(t)]$$

(28) $$w(t)=\left\{ \begin{array}{l} \frac{a_{3}}{t+10}+b_{3}\;\;0\leq t\;<60\\ \frac{a_{4}}{t+10}+b_{4}\;\;60\leq t\;\; \end{array}\right.\;\;\;$$

Other model parameters used in this example are shown in Table 1.

In Table 1, the selection of parameter values is to illustrate the effect of the four factors on the evacuation demand curves and conduct sensitivity analyses of the parameters. The values of $\gamma^{1}$, $\gamma^{2}$, and$\gamma^{3}$ are assumed to be the same, independent of any community. Those parameter values are assigned on the basis of reasonable and realistic evacuation scenarios. For an example, $\lambda_{0}$ = 0.25 means that on average, every four hours an evacuated person will contact one person inside of the community.

### 4.3. Model Results

In this example, the initial value $I_{1}^{1}(0)$ is assumed to be equal to 150 based on a report that around 400–500 people in Wanke Community (Community 1 in this case) have left their home before the evacuation order were issued [50]. Without data about the initial numbers of evacuated people in other communities, we cannot directly get other initial values for$I_{j}^{k}(0)$. In fact, the initial percentage of evacuated people in each community is mainly influenced by the risk level of the disaster. In general, the risk level is considered to be related with the distance to the risk source. So we assume that there is an inverse proportional relationship between the initial percentage of evacuated people in each community and a third power of the distance to the explosions source. The third power can also be substituted with first power or second power depending on how deep the distance is regarded to affect the initial percentage of evacuated people. In fact, the method above to set initial values is often used in many model solving cases [51].

With the analyses above, we can get the initial number of evacuated people in each community as follows:

(29) $$I_{j}^{k}(0)=\frac{\gamma^{k}}{\gamma^{1}}{\left(\frac{d_{j,0}}{d_{1,0}}\right)}^{3}\frac{Q_{j}^{k}}{Q_{1}^{1}}I_{1}^{1}(0)\;\;\;\;k=1,2,3;\;\;j=1,2,...,61$$

The time interval equals to one hour in this case. A simple numerical iteration algorithm is used to get the model results. This algorithm discretizes Equation (17) and calculates the increased percentage of each type of evacuated people in each community at the next time point based on the relevant cumulative percentage at present time point. With the Matlab software, the model results are shown in the following parts.

#### 4.3.1. Total Evacuation Demand Curve

**Figure 4:**
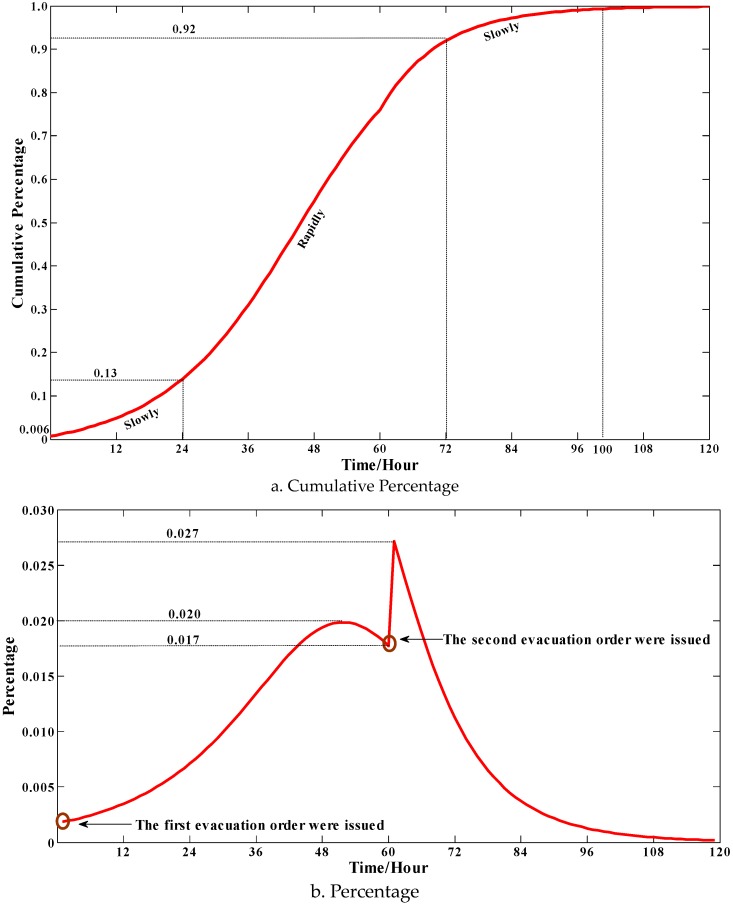
The total evacuation demand curve.

From Figure 4a, the percentage of evacuated people is just 0.6% (770) at *t* = 0 and t=0. However, the percentage increases rapidly from *t* = 0 to *t* = 72 and then the total evacuation demand rises slowly, and at *t* = 72 it approaches the peak. The whole total evacuation demand curve is like an S-curve and the increasing rule follows “slowly-rapidly-slowly”. The first “slowly” can be explained that the social influence power is weak because the total percentage of evacuated people is small at the initial stage. Similarly, at the final stage the total percentage of unevacuated people is also small, which can explain the second “slowly”.

Figure 4b shows the increased percentage of evacuated people in every hour. It is shown that the increased percentage of evacuated people in an hour has a “rise-decrease” trend as a whole. Specifically, the percentage is rising from 0.002 to 0.020 until *t* = 50 and then begins to drop. However, the percentage moves up sharply from 0.017 to 0.027 during *t* = 60 to *t* = 61, which can be explained because the issue of a second evacuation order accelerates the evacuation demand. After *t* = 61, the percentage is slowing down.

#### 4.3.2. Effect of Individual Characteristics

**Figure 5:**
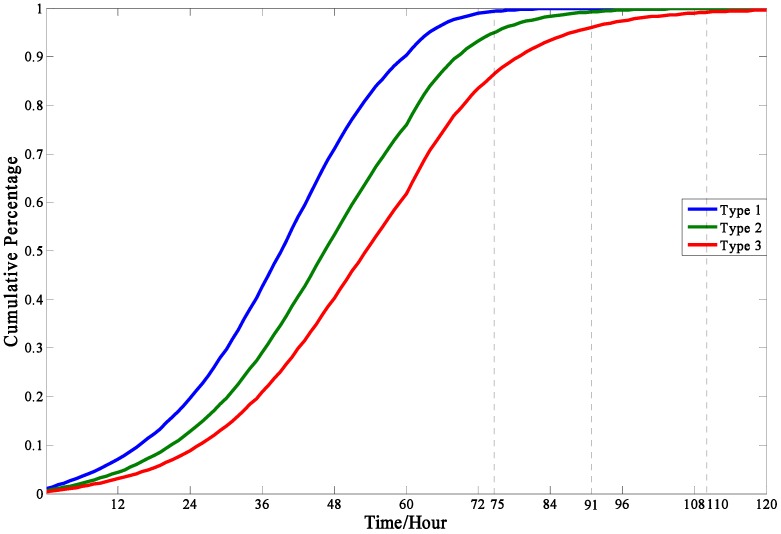
The evacuation demand curves in different types.

In the Section 2.2.1, people were divided into three types (“Impressionable”, “Neutral”, and “Standpat”) with the consideration of individual characteristics. So it is easily shown in Figure 5 that people in Type 1 are more likely to make an evacuation decision than that in Type 2 or Type 3, for people in Type 1 are more easily influenced by social influence, geographic location, and warning degree.

#### 4.3.3. Social Influence and the Effect of Geographic Location

**Figure 6:**
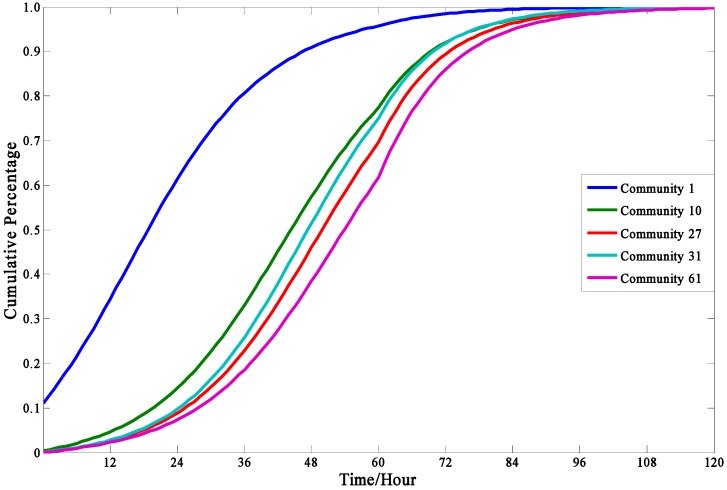
The evacuation demand curves in different communities.

In the Section 2.2.2., it is considered that the social influence on evacuation decisions is related with the influence function, the contact frequency function, and the risk level function, all of which are mainly related with the location of communities. So it is meaningful to compare the evacuation demand curves in different communities. Figure 6 shows five evacuation demand curves in Communities 1, 10, 27, 31, and 61. The geographic locations of them are shown in Table 2.

According to the Figure 6, it is obvious that the cumulative percentage of evacuated people in Community 1 is consistently higher than that the other communities all the time. It can be explained that Community 1 is closest to the explosion source among the five communities. Although the explosion source is slightly farther to Community 31 than Community 27, the cumulative evacuation demand in Community 31 is always less than that in Community 27, which is due to the weaker social influence in Community 27 (with only one adjacent community) or low contacts with other communities. This result is consistent with the conclusions in the previous research. Samiul Hasan [9] indicated that people in the community with few adjacent communities always have a lower propagation of disaster evacuation decisions. Moreover, it is pointed out by Perry [52] that the more the people have social contacts with other communities, the more likely that they will evacuate. The finding is significantly revelatory for the authorities to make specific evacuation plans. For example, due to the weak social influence in isolated communities, extra actions might be taken to accelerate evacuation process in those communities (i.e., enhancing the warning degree in this area).

#### 4.3.4. The Effect of Warning Degree

**Figure 7:**
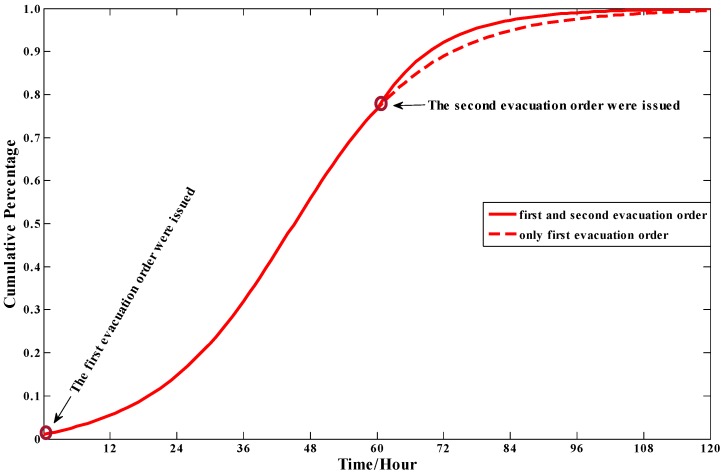
The total evacuation demand curve in different warning degrees.

During the evacuation process, the two evacuation orders were issued respectively at *t* = 0 and *t* = 60. From the Figure 7, it is clear that when the second evacuation orders were issued, the total demand increases more quickly than that without this evacuation order, which can be explained that the enhancing evacuation degree improves people’s perceived risk levels. This finding is meaningful in reality. The issue of warning information can be regard as a management strategy for the authorities, which can change the evacuation demand curves efficiently.

### 4.4. Sensitivity Analyses

According to the method in Section 3, we perform sensitivity analyses of parameters one by one. Firstly, a set of basic reference values and respective value ranges of parameters are given in Table 3. Then, from Equation (30), we get the sensitivity curves of parameters using the Matlab software.

It should be pointed out that we just analyze the sensitivity of parameter $\gamma^{1}$ instead of $\gamma^{2}$ and $\gamma^{3}$, which is reasonable because of the same role played in this model. It is same with $a_{3}$ and $b_{3}$.

**Figure 8:**
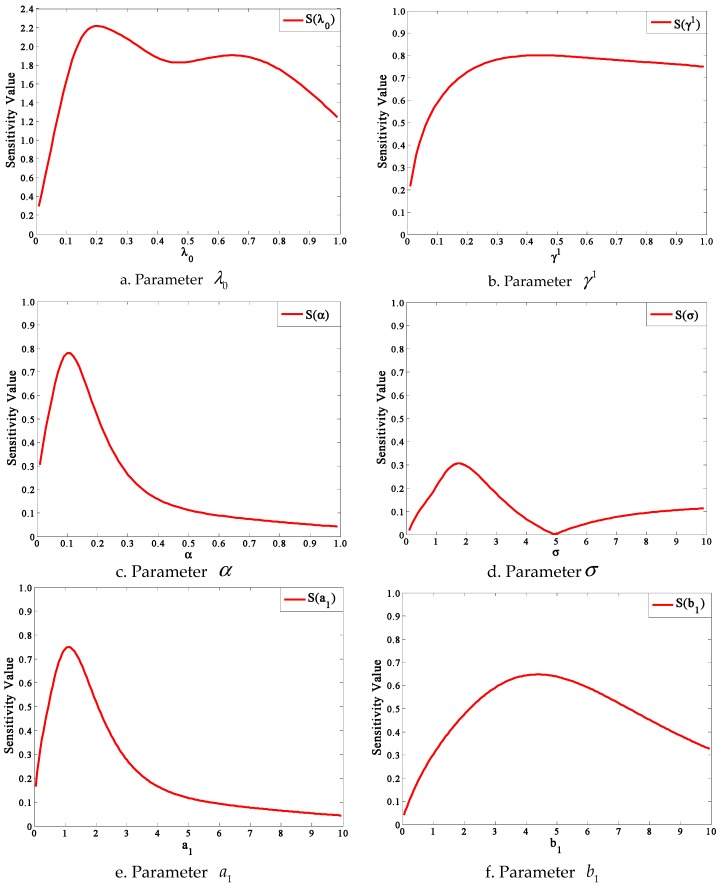

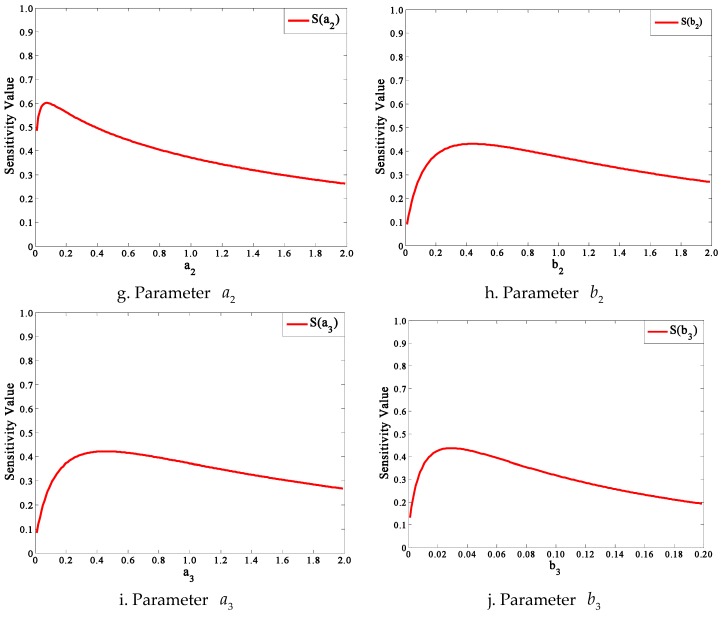
The sensitivity values of parameters in this model.

Figure 8 shows the variations of sensitivity with parameters in this model. In the most of the sensitivity curves about parameters, the sensitivity values increase rapidly first to the peaks and then slow down. Specifically, the sensitivity $S(\lambda_{0})$ has a sharp rise (from 0.3 to 2.2) at beginning and then decreases undulately with the parameter value increasing in the Figure 8a. This undulate trend of the sensitivity $S(\lambda_{0})$ is caused by the nonlinearity of parameter $\lambda_{0}$. The sensitivity $S(\gamma^{1})$ increases sharply until $\gamma^{1}=4$ and then begins to drop slowly in the Figure 8b. In the Figure 8c,e, the sensitivities S(α) and $S(a_{1})$ show similar trends (“increase rapidly−decrease sharply−decrease slowly”), which can be explained in that the two parameters have the same peculiarity in Equation (33). Similarly, parameters $b_{2}$ and $a_{3}$ have the same roles in Equation (33) where they are both coefficients about t which can account for the similar sensitivity curves in the Figure 8h. However, in the Figure 8d, the sensitivity S(σ) changes following “increase rapidly-decrease sharply-increase slowly” with the parameter value increasing.

**Figure 9:**
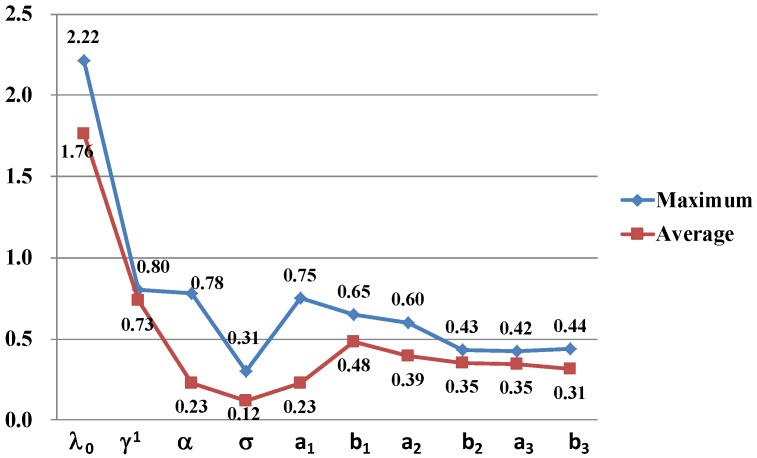
The maximum and average sensitivity values of parameters.

Figure 9 shows the maximum and average sensitivity values of parameters in respective value ranges. It is found that the sensitivity of parameter $\lambda_{0}$ is strongest among the 10 parameters based on the maximum or average sensitivity value, which means that the total evacuation demand curve is very sensitive with the contact frequency among people in risk areas over unit time. In contrast, the model is least sensitive with parameter σ, which means that the total evacuation demand curve is not sensitive with the shape of the contact frequency distribution curve between communities. It should be pointed out that the sensitivity results are based on a certain set of basic reference values of parameters. In other words, the sensitivity results may be different if we change reference values of parameters.

## 5. Discussion

Compared with models in previous studies, there are some advantages to this model. Firstly, it is clear to formulize the roles played by the most important factors in people evacuation decision making in this model. This model reveals the generating mechanism of evacuation demand curves from the view of social contagion and it can benefit authorities to adjust the evacuation demand as needed, which is not fully covered in previous models based on mathematical statistics like the S-curves [37]. Secondly, this model is developed on a group level instead of an individual level, which means that it is easier to get demand curves when a larger population is chosen. Moreover, the model framework is flexible for practical application. The flexibility reflects in two aspects. On the one hand, the model framework can be applied in various disasters just by some adjustment of the functions, such as the distance influence function in this model. For example, in poisonous gas dispersion, a second order parabolic partial differential equation can be chosen to describe the risk level influenced by geographic location and time. On the other hand, when a new factor is proofed to influence the curve, it can effectively be captured in this model only by changing or adding several variables.

In this paper, the “Tianjin Explosions” were used as a case to analyze the curves influenced by the four factors and perform the sensitivity analyses of the parameters in this model. The major conclusions of this study are conducted based on this case:

- The evacuation demand curve in this model is like an S-curve, in which the evacuation demand increasing rate starts increasing slowly, then rapidly, slowly again, and gradually closes to zero. This model result agrees with those obtained in mathematical statistics based on empirical data.
- The individual characteristics can dramatically influence people’s evacuation decision making and the cumulative evacuation demands of the “Impressionable” people are always greater than that of “Neutral” and “Standpat” people.
- The cumulative evacuation demands of people in isolated communities would be less than the communities with many adjacent communities, which can be explained in that the isolated communities lack social influence and people in these areas cannot easily get information on the real-time evacuation state of the society.
- A higher warning degree issued can enhance people′s perceived risk levels so as to significantly increase evacuation demand. Since the influence of warning degrees raised is imposed on the whole people in the risk areas, the authorities can accelerate or slow the evacuation process by changing the warning degree.
- In the sensitivity analyses of parameters, the model is more sensitive to the contact frequency among people over unit time than other parameters. To improve the prediction precision of evacuation demand curves, great attention should be paid to the contact frequency among people.

In this model, everyone would eventually evacuate, which, however, does not affect that the model proposed can work in the disaster evacuation scenes with varying severities. In the evacuation scenes where all people need to eventually evacuate, this model can effectively predict the evacuation demands in each community no matter the time interval. In the evacuation scenes where the emergency is not very severe, in this model, the demand curves may be very flat and a long time may have passed when all people evacuate. The curves can also reflect the evacuation demands in early days, which may be enough for the emergency authorities. In previous studies, researchers always estimate the evacuation demands in two steps [10,53,54]. The first step is to identify the region that needs to be evacuated and estimate the number of people to be evacuated. The second step is to determine the evacuation departure time or the evacuation demand curves. In this paper, we focus on the evacuation demand curves rather the whole evacuation demand estimation. There are many models to do the work in the first step like cross-classification type of trip generation model and logistic regression models. In future works, we will focus on how to combine these two steps into one model.

## 6. Conclusions

To prevent or at least minimize the losses caused by disasters, evacuation is regarded as an effective response. In the whole evacuation plan, estimating the disaster evacuation demand curves is a significant step which directly affects the evacuation performance. In this paper, we discussed the factors influencing the individual evacuation decision and summarized them into four parts. In the view of social contagion of decision making, we developed a model framework based on the SI model for formulizing the evacuation demand curves and examining the effects of the influencing factors on the curve shape. Using this model, we analyzed and illustrated the modeling results and performed the sensitivity analyses of the model parameters in the “Tianjin Explosions” case. Some interesting phenomena were founded and discussed, which is meaningful for authorities to make specific evacuation plans.

It should be pointed out that there were also some limitations in this model. In this paper, the concrete expressions of some functions are not given, such as the risk level function about location and time for different characteristics of disasters. When the appropriate data about time-dependent disaster evacuation demands were available, the models′ parameters should be calibrated. Therefore, we will focus on the concrete expressions of some functions and the parameter calibration for certain disaster scenarios in future works.

## Figures and Tables

**Table 1:** Values of parameters in this case.

**Parameter**	$\lambda_{0}$	$\gamma^{k}$	λ(d)	h(d)	f(d,t)	w(t)
		$\gamma^{1}$ $\gamma^{2}$ $\gamma^{3}$	α σ	$a_{1}$ $b_{1}$	$a_{2}$ $b_{2}$	$a_{3}$ $b_{3}$ $a_{4}$ $b_{4}$
**Value**	0.25	0.5 0.3 0.2	0.01 1	1 0.1	0.01 0.01	0.01 0.001 0.2 0.01

**Table 2:** The geographic locations of the five communities.

Community	Distance to the Explosion Source (km)	Surrounding Community
1	0.67	2, 4
10	2.06	8, 9, 16
27	4.12	41
31	4.21	23, 30, 38, 48, 50
61	6.10	60

**Table 3:** The values of parameters and some results in sensitivity analyses.

Parameter	Basic Reference Value	Value Range	Maximum Sensitivity	Average Sensitivity
$\lambda_{0}$	0.25	(0–1)	2.2165	1.7619
$\gamma^{1}$	0.5	(0–1)	0.8012	0.7344
α	0.1	(0–10)	0.7795	0.2301
σ	1	(0–10)	0.3051	0.1168
$a_{1}$	0.1	(0–10)	0.7493	0.2281
$b_{1}$	0.1	(0–10)	0.6477	0.4832
$a_{2}$	0.1	(0–2)	0.6005	0.3940
$b_{2}$	0.02	(0–2)	0.4308	0.3522
$a_{3}$	0.1	(0–2)	0.4224	0.3470
$b_{3}$	0.002	(0–0.2)	0.4371	0.3131
